# Longitudinal Changes in Physical Activity Level, Body Mass Index, and Oxygen Uptake Among Norwegian Adolescents

**DOI:** 10.3389/fpubh.2018.00097

**Published:** 2018-03-29

**Authors:** Pål Lagestad, Roland van den Tillaar, Asgeir Mamen

**Affiliations:** ^1^Nord University, Levanger, Norway; ^2^Norwegian School of Health Sciences, Oslo, Norway

**Keywords:** youth, physical activity level, physical fitness, body mass index, longitudinal

## Abstract

Several studies have investigated activity levels among adolescents, but no study has examined longitudinal changes in physical activity (PA) level, body mass, and oxygen uptake among the same adolescents from the age of 14 to 19 years. The present study examined data from a research project that included a group of randomly selected students (*N* = 116) with objective measurements of PA (accelerometer data), self-reported PA level, and body mass and oxygen uptake during a 5-year period. The results show a significant decrease in the accelerometer-based PA level over time, from age 14 to 19. At 14 years of age, the minutes of moderate and/or vigorous PA was 66.7 min·day^−1^, but was less than half, at only 24.4 min·day^−1^, at 19 years of age. The self-reported activity data show a decrease in girls’ general activity level over time, while boys’ activity level during school breaks decreased strongly during the period: at age 14, 61% of the boys were classified as active, while at age 19, only 11% were physically active. Furthermore, body mass index increased during the period for both genders, while oxygen uptake decreased. Since both BMI and maximal oxygen uptake are important risk factors for future CVD, these findings point toward the importance of maintaining a high activity level during childhood and adolescence, in order to keep fit later in life.

## Introduction

The beneficial effect of physical activity (PA) on cardiorespiratory fitness (CRF) is well documented in children, adolescents (1), and adults (2). The presence of risk factors for diabetes, hypertension, site-specific cancers, dyslipidaemia, cardiovascular morbidity, and bone health are inversely related to PA levels (3–6).

However, research indicates that today’s youth across the world are becoming less physically active than previous generations. In Norway, Dalene et al. (7) found a reduction in minutes of moderate and/or vigorous physical activity (MVPA) in both 9- and 15-year-old children between 2005 and 2012, and also a study by Kolle et al. (8) indicates that participation in PA declines during adolescence in Norway. This study showed that while 86% of 9-year-old boys and 70% of 9-year-old girls met PA guidelines, the figures at 15 years were only 58% for boys and 43% for girls. A significantly lower percentage of girls than boys met the guidelines at every age level, and girls also had a lower activity level than boys at every age level. Belton et al. (9) found that the majority of adolescents were not accumulating the 60 min of daily PA that is recommended for health and that 99.5% of children did not achieve the fundamental movement skill proficiency expected for their age. A general worldwide decline in activity level among adolescents is indicated by research from other countries (10–16).

To correctly describe the development of PA among adolescents, longitudinal studies should be undertaken. However, while cross-sectional studies are common, longitudinal studies in this field are limited. Ball et al. (11) presented data from the Children Living in Active Neighborhoods study on accelerometer-measured and self-reported PA and sedentary behavior in children 6–12 years old at study start. Three years later, MVPA levels had declined among both girls and boys. Telama and Yang (15) found a remarkable decline in frequency of PA and sport participation after the age of 12, and a 5-year follow-up study of 12- to 13-year-old adolescents found that the prevalence of participation in most activities declined over these 5 years (12). Kimm et al. (17) noticed a steep decline in both self-reported and accelerometer-measured PA for girls from age 10 to 19. Trang et al. (16) reported a decline in MVPA levels of Vietnamese children over 5 years, measured both objectively and by self-report.

Research suggests that there appears to be a decline also in young people’s aerobic performance on a global scale, as shown in Norway (18), in other Western countries, and in Asia (19–21). A decrease in VO2peak relative to body weight over the whole age range from 12 to 36 years of age has been found (22). Boys’ VO2peak values were higher than those of girls (22–24).

The previous discussion indicates a decline in activity level during adolescence. However, longitudinal studies in this research area are limited, and there is a lack of research on longitudinal development of PA level, body mass index, and oxygen uptake among adolescents from the age of 14 to 19. The aim of this study was to provide new evidence of longitudinal changes in PA level, body mass index, and oxygen uptake among adolescents over a 5-year period, from age 14 to 19 years.

## Materials and Methods

### Design

To examine the research question, a longitudinal study was used, with repeated measures among the same adolescents from the age of 14 to the age of 19. Peak oxygen uptake during treadmill running, weight, height, and PA levels (both accelerometer and self-reported data) were recorded during the 5-year period. Approval to use the data and conduct the study at the high school in question was given by the Norwegian Centre for Research Data (NSD) and the Norwegian Regional Committee for Medical and Health Research Ethics (ID: 488715, 2014).

### Subjects

Six classes (three groups of two classes) in two schools from a small town in central Norway were randomly selected for the study. Of the 144 students, 124 students agreed to participate in the study, but only 116 students took the pre-test (age 14 ± 0.5 years, weight 54.2 ± 10.9 kg, height 1.63 ± 0.08 m at the start of the study). The distribution of boys and girls was relatively equal in the sample (61 boys and 55 girls), as well as the distribution of students living in urban and rural areas. The number of students who had valid accelerometer data during the data collection was 68 at 14 years, 50 at 16 years (there were no accelerometer measures at 15 years), 68 at 17 years, 37 at 18 years, and 22 at 19 years (the final year of high school). The reasons for invalid data were dropout because of illness, injury, pregnancy, or moving away, in addition to some students not turning on the accelerometer. Only eight students had valid accelerometer data for all five measurement times.

Completed data on self-reported PA were as follows: 14 years: 106 (58 boys and 48 girls), 15 years: 103 (57 boys and 46 girls), 16 years: 105 (58 boys and 47 girls), 17 years: 82 (48 boys and 34 girls), 18 years: 65 (41 boys and 24 girls), and 19 years: 86 (41 boys and 45 girls). Forty-nine students (29 boys and 20 girls) had completed the questionnaire and reported their PA level in leisure time during all six test-years and were included in the analysis.

Completed data on self-reported PA in school breaks were as follows: 14 years: 104 (56 boys and 48 girls), 15 years: 101 (56 boys and 45 girls), 16 years: 104 (58 boys and 46 girls), 17 years: 79 (47 boys and 32 girls), 18 years: 65 (41 boys and 24 girls), and 19 years: 79 (39 boys and 40 girls). Forty-two students (28 boys and 14 girls) had completed the questionnaire and reported their PA level in school breaks during all six test years and were included in the analysis.

The subjects were informed orally and in writing about the aim and methods of the study before participation. Written consent was signed each year by the parents (at students’ age of 14 and 15) and by the students (at 16–19 years) according to the ethical regulations for research. Approval to use the data and conduct the study was given by the NSD and the Central Norway Regional Committee for Medical and Health Research Ethics (REK).

### Procedures

Before the tests, the students had been given information about the conditions for preparation before the test (avoid strenuous exercise the day before, eat 2–3 h before the test, no more than a “light” breakfast, a PE class before the test was possible, but only light activity, and a 15-min warm-up just before the test). An inclination of 10.5% was used on the treadmill according to the test procedures. This was to ensure that running technique was not a limiting factor for maximum oxygen uptake. Before the test, the students were asked how much they exercised. Girls who did not exercise regularly or were overweight, started with a velocity of 4 km/h, those who exercised 1–2 times a week started with a velocity of 5 km/h, while those who exercised three or more times a week, started with a velocity of 6 km/h. For boys, the same categories were used, but with 1 km/h higher velocity. The velocity on the treadmill was increased by 1 km/h every minute, except sometimes at the end of the test, where the velocity was increased by only 0.5 km/h. The criterion for the highest maximal oxygen uptake was a flattening/decrease of the O_2_ curve with increasing velocity (RER > 1.00). The average of the two highest successive measurements was recorded as maximum oxygen uptake. The test had a duration of 5–6 min. The oxygen uptake measurements were carried out on the treadmill, using a Woodway S5 (Woodway Inc., Waukesha, WI, USA) and OxyCon Pro (Erich Jaeger GmbH, Hoechberg, Germany).

Height was measured with a stadiometer (Kawe, NorEngros, Oslo, Norway), permanently connected to the wall. The subjects did not wear shoes, and height was converted to the nearest centimeter. Weight was measured using Seca Digital Weight Scales (Seca Gmbh & Co., Germany, Model 877, accuracy of 0.1 kg). Body mass index was calculated by dividing weight (kilograms) by height (centimeters) squared and then multiplying the result by 10,000, in accordance with international standards (25).

The participants followed the test protocol by answering a questionnaire containing questions used with adolescents by Aspvik et al. (26), including one about activity level during school breaks. Possible responses were not active and active. The questionnaire also included a question about PA level in the past 4 weeks: when you think about your PA in the past 4 weeks, how often did you participate in sport/exercise or other PA with such an intensity that you breathed fast, sweated, or your heart beat fast for 20 min? Alternative responses were never, less than once a week, once a week, 2–3 days a week, and most days in the week. Finally, the subjects were also asked if they were active or inactive at break time at school. The students spent around 40 min on the measurements and answering the questionnaire.

At the end of the test protocol, the students were given an Actigraph GT1M accelerometer to measure their activity level (MVPA). According to the procedures of Norwegian population studies of adolescents (8), persons should wear the accelerometer in a belt on their right hip for 1 week, only taking it off before going into water and going to sleep at night. After 1 week, the accelerometers were collected, and the data were downloaded into the programme Actilife v6.13.3 (ActiGraph, LLC, Pensacola, FL, USA) and analyzed. A 10-s epoch was used. Following Kolle et al. (8), missing data were defined as continuous periods of 20 min or more with no counts. All activity during the night (24:00–06:00) was deleted in accordance with the same test protocol. Furthermore, each day had to include at least 480 min of activity to be valid, and each student had to have at least two valid days to be included in the analysis. The cut-off for moderate intensity was set to 2,000 counts, in accordance with the test protocol of Kolle et al. (8).

The measurements took place during April and May of each year when the students were 14, 15, 16, 17, 18, and 19 years of age. All tests were carried out by the same test leader, with the same equipment, with the same procedures, and in the same room.

### Statistical Analysis

**Figure 1:**
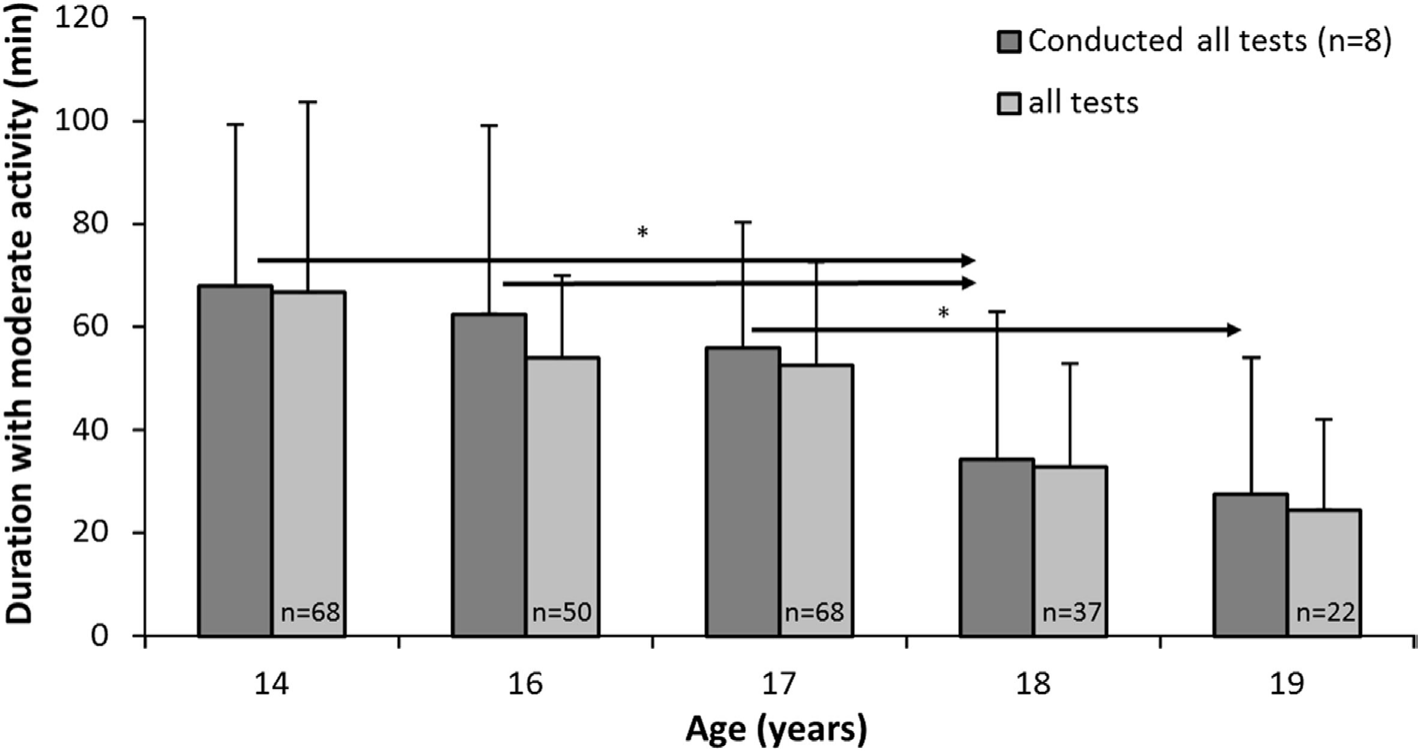
Minutes of moderate or vigorous physical activity (PA) (daily) among subjects with valid data on all five tests (*n* = 8) and for all subjects tested on each test day. * indicates a significant decrease in PA between these test years and later years at a *p* < 0.05 level, as indicated by the right-pointing arrow.

Because of considerable dropout on the accelerometer data, the results were presented using group data, including subjects with valid data at each measurement time, but individual data on subjects with valid data at every measurement time were also used (Figure 1). A repeated measures ANOVA was used on the individual data to analyze the development of the PA level (accelerometer data) during the five measurements, but also the development of body mass index and oxygen uptake. Effect size was evaluated with η^2p^ (Eta partial squared), where 0.01 < η^2^ < 0.06 indicates a small effect, 0.06 < η^2^ < 0.14 a medium effect, and η^2^ > 0.14 a large effect (27).

The conditions for a parametric test were not fulfilled, and Friedman’s non-parametric test was used to examine whether the students self-reported PA changed during the six measurement times, while the Mann–Whitney *U*-test was used to analyze differences between genders, according to PA level. The Wilcoxon test was used to analyze differences from year to year for each gender according to self-reported PA at leisure time and in school breaks. An independent *t*-test was also used to examine differences between the 8 subjects with valid accelerometer data at all six measurement times and the other 60 subjects who only had valid data at 14 years of age. A chi-square test was used to examine the association between the 49 with valid self-reported PA data at all six measurement times (included in the analysis) and the 55 who had valid data at 14 years, but not for all of the other five tests. Statistical significance was set at *p* ≤ 0.05. SPSS version 22 was used to perform the analysis.

## Results

Figure 1 shows the development of activity level as measured by accelerometer of the students who took all the tests, but also of all students, because only eight students had valid accelerometer data during the period. The analysis of the students who took all five tests showed a significant decrease in accelerometer-based PA levels over time from 14 to 19 years (*F*_4,28_ = 4.922, *p* = 0.004, η^2^ = 0.413, 1 − β = 0.923). At 14 years of age, the MVPA level was 66.7, while it was only 24.4 at 19 years of age, i.e., less than half. *Post hoc* tests showed that the MVPA level dropped significantly from 14 to 18 years (mean dif. = 33.9 MVPA, 95% CI = 2.4–65.5, *p* = 0.039) and from 14 to 19 years (mean dif. = 42.2 MVPA, 95% CI = 16.5–67.9, *p* = 0.006). The MVPA also decreased significantly from 16 to 18 years (mean dif. = 21.2 MVPA, 95% CI = 4.3–38.1, *p* = 0.021), from 16 to 19 years (mean dif. = 29.4 MVPA, 95% CI = 9.5–49.3, *p* = 0.010), and from 17 to 19 years (mean dif. = 28.1 MVPA, 95% CI = 0.9–55.3, *p* = 0.045).

Further analysis showed that there were no significant differences (*p* > 0.005) between subjects with valid data on all five tests (*n* = 8) and all subjects tested on each test day, at each of the five measurement times.

**Figure 2:**
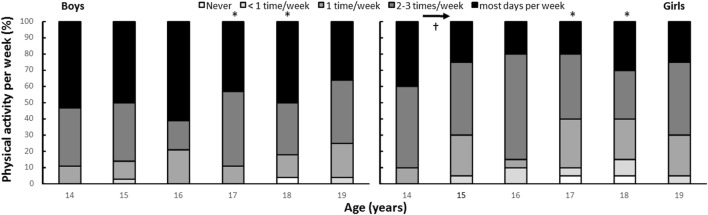
Self-reported physical activity (PA) per year by gender (boys: *n* = 29, girls: *n* = 20). * indicates a significant gender difference at this age at a *p* < 0.05 level. ^†^ indicates significantly higher self-reported PA compared with all other ages.

Self-reported activity level at 14 to 19 years of age, measured by questionnaire, decreased significantly during the period among girls ($\chi_{2}^{2}$ = 11.38, *p* = 0.044), but not among boys ($\chi_{2}^{2}$ = 5.74, *p* = 0.332). Further analysis showed that the activity level of girls was significantly higher at 14, compared with the other ages (*Z* ≤ −2.09, *p* ≤ 0.037, Figure 2). Boys reported a higher activity level than girls at the age of 14 and 18. However, there were no gender differences at the other four measurement times (*p* > 0.005).

According to self-reported activity levels at the age of 14, there was no significant association (*p* > 0.005) between the 49 subjects with valid data on the six tests and the 56 subjects who took the pre-test at 14 years but dropped out ($\chi_{4}^{2}$ = 5.98, *p* = 0.200).

**Figure 3:**
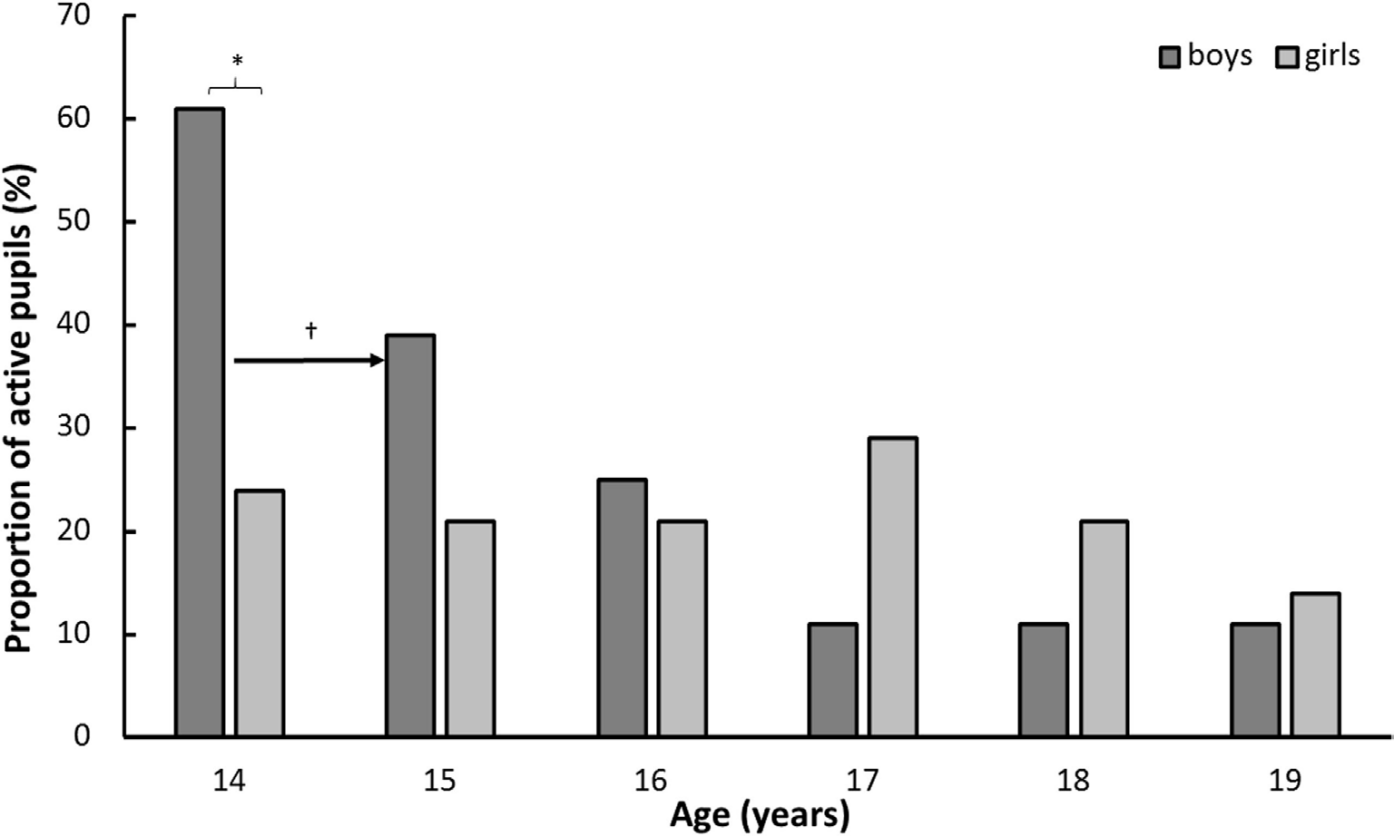
Proportion of students physically active during school breaks by gender and year (boys: *n* = 28, girls: *n* = 14). * indicates a significant gender difference at this age at a *p* < 0.05 level. ^†^ indicates a significant difference between this age and a later age at a *p* < 0.05 level, as indicated by the right-pointing arrow.

A reduction in physically active students during school breaks was found with increasing age (χ^2^ = 25.2, *p* < 0.001). However, *post hoc* analysis showed a reduction in activity levels during this time among boys (χ^2^ = 36.5, *p* < 0.001), but not among girls (χ^2^  = 1.0, *p* = 0.963). Furthermore, a higher activity level was reported by boys than girls at the age of 14 (*Z* = −3.00, *p* = 0.003). In addition, boys’ PA only reduced from the age of 14, to the age of 15 (*Z* ≥ 3.13, *p* ≤ 0.037, Figure 3).

For PA in school breaks at the age of 14, statistical analysis showed a relationship between the 42 included in the analysis and the 37 measured in 8th grade who later dropped out (χ^2^ = 5.26, *p* = 0.022). While 47.6% of the students included in the analysis reported PA in school breaks in 8th grade, this was reported by 25.8% of the dropout students.

**Figure 4:**
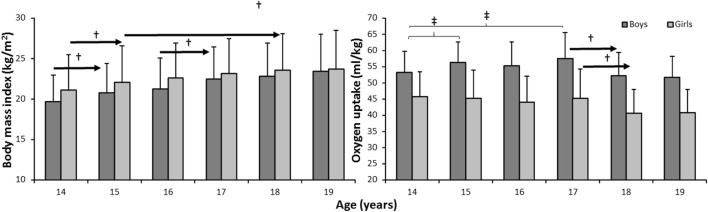
Development of body mass and oxygen uptake over the 6 years by gender (boys: *n* = 29, girls: *n* = 20). ^†^ indicates a significant difference for this gender at a *p* < 0.05 level between this age and later ages, as indicated by the right-pointing arrow. ^‡^ indicates a significant difference for this gender between these two ages at a *p* < 0.05 level.

Body mass index increased significantly, while oxygen uptake decreased over the years (*F* ≥ 26.8, *p* < 0.001, η^2^ ≥ 0.36, Figure 4). A significant gender effect was found for oxygen uptake (*F* = 3.8, *p* = 0.002, η^2^ = 0.08), where boys had a higher oxygen uptake than girls at all ages. However, there was no significant gender effect in body mass index (*F* = 1.6, *p* = 0.146, η^2^ = 0.036). *Post hoc* comparison showed significant increases from the age of 14 to 15 for body mass index for both genders and for girls from age 15 to 18, while BMI for boys increased from age 16 to 17 (Figure 4). Oxygen uptake decreased significantly from the age of 17 to 18 for girls, while for boys, first an increase in oxygen uptake from 14 to 17 was found, but after the age of 17 it decreased significantly to the level at the age of 14 (Figure 4).

## Discussion

We found that from age 14 to 19, accelerometer-measured MVPA levels declined for both boys and girls. This was valid both for the eight students who completed all measurements (from 67 to 24 min·day^−1^) and the cross-sectional measurements (from 65 to 22 min·day^−1^). This decrease seems to be linear. The analysis is only based on eight adolescents because of dropout, but Figure 1 clearly shows that the numbers are almost the same for all subjects tested on each test day (*n* varies from 22 to 68). Self-reported frequency of PA per week did not change significantly for boys, but did significantly decrease for girls. The self-reported activity data showed that boys reported a higher activity level than girls at the age of 17 and 18, but there were no gender differences at the other four measurement times.

Another main finding was the significant reduction in the proportion of boys who reported being physically active during break time at school with increasing age. While 61% of boys reported being active at the age of 14, only 11% of boys reported this at the age of 19. The findings showed a less clear pattern for the girls, but at age 19, the girls did participate less in break time PA compared to age 14. Furthermore, self-reported activity level during school breaks dropped for boys and directly measured aerobic power showed an increase from 14 to 17 years for boys, but then decreased to levels below those of 14 years. The aerobic power of girls declined modestly, but statistically significantly throughout the period. BMI increased for both genders throughout the period.

These results support other research among adolescents who indicate a decrease in PA levels for Norwegian adolescents (8) and a similar decrease worldwide (10–16). Only a few studies have explored longitudinal development of PA among children and adolescents. Dalene et al. (7) found decreased PA for both boys and girls from age 9 to 15. For both boys and girls, the change was highly significant for overall PA (lower at age 15), sedentary time (higher at age 15), light PA (lower at age 15), and moderate to vigorous PA (lower at age 15). Ortega et al. (28) investigated a group of 9- and 15-year-old boys and girls from Sweden and Estonia in 1998 and again 6–10 years later. They found also an overall decline in MVPA of 30 min·day^−1^. The reduction in MVPA was greater in Sweden than Estonia and for boys compared to girls. The 9-year-olds from 1998 showed a greater decline in MVPA than those who were 15 years old in that year. Kimm et al. (17) followed boys and girls for up to 10 years. PA decreased, both from self-report and objectively (accelerometer-measured activity as counts·day^−1^ declined by 21%) measured PA. Wei et al. (29) followed Chinese children for 8 years, showed just a small reduction in MET h·week^−1^, from 50 to 47, while Brooke et al. (30) found that accelerometer-measured activity (counts·min^−1^) and MVPA (min·day^−1^) decreased after 4 years. Boys were significantly different from girls in reduction of number of activities and MVPA, but no other differences were found between the genders. Francis et al. (31) studied tracking of PA from age 6 to 15, and found both MVPA and vigorous PA declined for both sexes, although the boys had an increase in the first years, but then dropped below initial levels. Total PA (counts·min^−1^) decreased steadily for boys and girls throughout the period.

Overall, longitudinal studies from different youth population groups agree that PA declines with increasing age, as we also found (7, 13, 17, 30–34). However, previous longitudinal studies have examined PA levels among children up to 15 years of age, while our study examined changes in activity levels among adolescents from 14 to 19 years.

We found that the self-reported activity to some extent matched the objective measurements, but the self-reported development of the boys deviated somewhat from the accelerometer measurements. It is usual to find some divergence between the two, as shown in the present study, but self-report is still recognized as a valid method to quantify PA (35–40), even though some studies find low correlation (41). One interesting finding on the validity of self-report questionnaires is the effect of education level on the results; here, people with low education levels give less valid information (42, 43).

It may be argued that PA during break time at school only represents a minor part of an adolescent’s day; nevertheless, the finding of a decrease in this activity, and the decrease in PA levels in general, gives cause for concern. The decrease in PA with increasing age is probably the result of many different factors. Type and level of PA change with age (30, 32, 44–47). Also, the social norms for PA might change, and this might influence activity level and also self-reported activity (48–51). The amount of schoolwork increases with the student’s age, thus reducing the time for PA. Interests also change, and the number of interests or activities decreases, sometimes to only one. With increasing age, students are more likely to use motorized transportation, which may explain some of the reduction reported. Thus, to counteract this negative development, society at large must react in a variety of ways.

The results showed that BMI increased during the period, but there was no significant gender effect in BMI. BMI increased from 14 to 15 in both genders, from 15 to 18 in girls, and from 16 to 17 in boys. In agreement with other studies (52–54), it is appropriate to highlight the negative effect of this increase, in relation to overweight and obesity as negative health factors. The results also showed that oxygen uptake decreased over the years, another negative finding in terms of health. High oxygen uptake (CRF) is closely connected to positive health-related factors, being associated with cardiovascular morbidity and mortality, and the prevention of cardiovascular diseases, such as diabetes, hypertension, site-specific cancers, bone health, and selected dyslipidaemias (3–6). Poor CRF is strongly associated with cardiovascular diseases in children, and this relationship is stable across countries, age, and gender (3).

### Strengths and Limitations of the Study

The major strength of the study is that it was based on a longitudinal design with the same participants, employing the same questions and tests every year, performed in the same room, and using the same test procedures, test equipment, and test leader for all of the six test measurements. Furthermore, many of the variables, such as VO2peak, overweight, and gender, are based on high-quality standard procedures. To the best of our knowledge, this is the first longitudinal study measuring adolescents’ PA levels from 14 to 19 years of age. Furthermore, the use of measurements such as accelerometers has the advantage of decreasing subjectivity (55) and eliminating bias, such as social desirability and recall problems (56). However, there are several limitations to the study. The number of participants was somewhat low. Furthermore, there was a huge dropout of students with accelerometer data and few students with valid data on every test, which reduces the potential impact of the results. However, analysis showed that there were no significant differences between subjects with valid accelerometer data and self-reported PA data at each measurement time and those without valid data on every measurement occasion.

## Conclusion

The longitudinal accelerometer data show a substantial decrease in MVPA levels (from 67 until 24) during the 5-year period from age 14 to 19. A substantial decrease in the proportion of boys who reported being physically active in school breaks during the period (from 61% until only 11%), underpins the reduction in activity level. Furthermore, the results showed that body mass index increased significantly during the same period, while oxygen uptake decreased significantly. These negative findings in relation to activity level, body mass index, and oxygen uptake are partly supported by other research, and they are particularly negative in a health perspective. The results indicate the importance of maintaining adolescents’ PA levels during youth, in order to reduce the negative trends in body mass index and oxygen uptake in adolescence.

## Ethics Statement

The subjects were fully informed about the protocol before participating in this study, and a written informed parental consent was obtained. Approval to use the data and conduct the study was given by the Norwegian Social Science Data Services (NSD) and the Norwegian ethical regional comité.

## Author Contributions

PL has contributed to design and methods, writing the introduction, methods, discussion, and the conclusion. Furthermore, a critical review of all the text during several numbers of the article and rewriting of the text. RT has contributed to methods and some minor contribution to writing the introduction, discussion, conclusion, and a critical review of all the text during several numbers of the article and rewriting of the text. AM has contributed to writing the introduction, discussion, and conclusion. Furthermore, a critical review of all the text during several numbers of the article and rewriting of the text.

## Conflict of Interest Statement

The authors declare that the research was conducted in the absence of any commercial or financial relationships that could be constructed as a potential conflict of interest.
